# Vocal cord dysfunction: a review

**DOI:** 10.1186/s40733-015-0009-z

**Published:** 2015-09-22

**Authors:** Neha M. Dunn, Rohit K. Katial, Flavia C. L. Hoyte

**Affiliations:** 1 National Jewish Health, University of Colorado, Denver, CO USA; 2 National Jewish Health, Denver, CO USA

**Keywords:** Vocal cord dysfunction, Paradoxical vocal fold movement, Vocal cord, Asthma-comorbidity

## Abstract

Vocal cord dysfunction (VCD) is a term that refers to inappropriate adduction of the vocal cords during inhalation and sometimes exhalation. It is a functional disorder that serves as an important mimicker of asthma. Vocal cord dysfunction can be difficult to treat as the condition is often underappreciated and misdiagnosed in clinical practice. Recognition of vocal cord dysfunction in patients with asthma-type symptoms is essential since missing this diagnosis can be a barrier to adequately treating patients with uncontrolled respiratory symptoms. Although symptoms often mimic asthma, the two conditions have certain distinct clinical features and demonstrate specific findings on diagnostic studies, which can serve to differentiate the two conditions. Moreover, management of vocal cord dysfunction should be directed at minimizing known triggers and initiating speech therapy, thereby minimizing use of unnecessary asthma medications. This review article describes key clinical features, important physical exam findings and commonly reported triggers in patients with vocal cord dysfunction. Additionally, this article discusses useful diagnostic studies to identify patients with vocal cord dysfunction and current management options for such patients.

## Introduction

Vocal cord dysfunction (VCD) is a term that refers to inappropriate adduction of the vocal cords during inhalation and sometimes exhalation [1]. It is a functional disorder that serves as an important mimicker of asthma. Concomitant vocal cord dysfunction and asthma are seen in a high degree of patients, up to 50 % of patients in some studies [2]. Recognition of vocal cord dysfunction in patients with asthma-type symptoms is often missed and can be a barrier to adequately treating patients with uncontrolled respiratory symptoms.

## Review

### Historical background

Vocal cord dysfunction was first described clinically in 1842 as dysfunction of the laryngeal muscles sometimes seen in hysterical women [3]. This condition was first visualized during laryngoscopy in 1869 by MacKenzie, who made the diagnosis in “hysteric” patients [4]. In 1902, Sir William Osler described VCD as a disorder affecting both the inspiratory and expiratory phases of the respiratory cycle in the textbook *The Principles and Practice of Medicine* [5]. VCD was next described in the medical literature 70 years later, in 1974, by Patterson and colleagues in a 33 year old woman with 15 hospitalizations for what they termed “Munchausen’s stridor” [6].

Since then, more than 70 terms have been used to describe abnormal movement of the true vocal cords. Today, the two most commonly encountered terms in medical literature are paradoxical vocal fold motion (PVFM) and vocal cord dysfunction. For the purpose of this article, we will use the term vocal cord dysfunction (VCD) to refer to the group of conditions that encompasses all of these terms.

## Clinical presentation

The true incidence of VCD is unknown, but is likely underappreciated in clinical practice. VCD was initially only thought to exist in the context of psychological illness or hysteria; however, over the past several decades, it has been recognized to occur outside of psychological illness and affect a broader patient base [1]. Brugman and colleagues investigated 1530 patients with VCD and found that 65 % were adults above 19 years of age, with a broad overall age range from 0.02 to 82 years. The median age range was 36.5 years in adults and 14 years in pediatric patients [7]. In addition, there tends to be a female predominance among patients with VCD. Brugman and colleagues found a 3:1 female predominance among their patients, while Morris and colleagues studied 1161 patients in another literature review and found a 2:1 female predominance [8].

The clinical presentation of vocal cord dysfunction is widely variable, ranging from no symptoms to mild dyspnea to acute-onset respiratory distress that can mimic an asthma attack [9]. Often, symptoms are periodic and have been refractory to prior prescribed medical therapy, such as asthma medications [1]. In a review of 1020 patients with VCD, Morris and Christopher found that symptoms were chronic in 860 patients (85 %) and acute in 151 patients (15 %) [10]. Patient-reported symptoms include air hunger, sensation of choking, chest tightness, chest pain, difficulty swallowing, globus sensation, intermittent aphonia or dysphonia, neck or chest retractions, fatigue and throat clearing. Many of these sensations can elicit fear, panic and anxiety, which can further worsen respiratory symptoms [1]. Many studies have described patients with VCD who have concomitant cough [11, 12]. One theory by Vertigan and colleagues proposed that chronic cough and VCD are different manifestations of a single underlying condition. They proposed a model of chronic cough and VCD on a continuum with pure cough at one end and pure VCD at the other with some combination of the two in the middle [13].

Patients with VCD are often misdiagnosed as having refractory asthma, which can lead to increased health care costs. Newman and colleagues studied 95 patients with VCD and found that these patients were misdiagnosed for an average of 4.8 years before being diagnosed with VCD. During this time, they were treated with medications for severe asthma, sometimes including daily prednisone, and required multiple ER visits, hospitalizations, and even intubation in 28 % of patients [11]. Traister and colleagues performed a study comparing 59 patients with asthma, 43 patients with asthma and VCD and 89 patients with VCD alone. They found that 42.4 % of all VCD subjects had been previously misdiagnosed with asthma for an average of 9 years. Those patients with coexisting VCD and asthma or asthma alone had increased health care usage compared to patients with VCD alone. Interestingly, patients with VCD alone who were misdiagnosed as having asthma had increased medication and health care usage compared to patients with VCD who were not given the diagnosis of asthma. This suggests that the main morbidity associated with VCD may lie in its ability to mimic asthma [14].

Physical examination can help to differentiate patients with VCD or asthma. Patients often point to or grab their throat when describing their respiratory symptoms [15–17]. Rather than helping symptoms, patients often report that metered dose or powder inhalers can trigger or exacerbate symptoms, whereas nebulized medications tend to provide relief [16]. During an acute attack, VCD often presents with stridor, tachypnea, hoarseness, dysphonia, cough, tugging of the neck or upper chest muscles and a look of anxiety or distress [17]. A patient’s noisy breathing can be reported on physical examination as “stridor” or “wheeze”. Patients may appear to be in extremis during an episode and may have complaints out of proportion to objective findings [18]. Many case reports have described that patients with VCD who require intubation are easy to ventilate, with quick resolution of symptoms and normal airway pressures, followed generally by extubation within 24 h [18].

The differential diagnosis for VCD is broad and includes any disorder with episodic dyspnea, cough and wheezing. There are many mimickers of VCD, with asthma historically on the top of the list. A broad differential is listed in Table 1 and is important as VCD can often mimic or coexist with many of these other conditions [1, 17].

**Table 1:** Differential diagnosis of laryngeal movement disorders [17, 24]

VCD	
Psychogenic	Somatoform disorder, conversion disorder, abuse, anxiety disorder, depression, Munchausen syndrome, malingering
Exercise	Exercise
Irritant	Extrinsic (chemical irritants, olfactory stimuli)
	Intrinsic (GERD, laryngopharyngeal reflux rhinitis/post nasal drip, sinusitis)
Laryngospasm	Intubation, airway manipulation, IgE mediated, nocturnal aspiration
Vocal cord paresis/paralysis	Prolonged intubation, recurrent laryngeal or vagus nerve damage during chest or thyroid surgery, idiopathic
Infectious	Epiglottis, bronchiolitis, laryngotracheobronchitis (croup), laryngitis, pharyngeal abscess, diphtheria, pertussis, laryngeal papillomatosis
Rheumatologic	Rheumatoid cricoarytenoid arthritis, relapsing polychondritis, laryngeal sarcoidosis
Neoplastic	Head and neck malignancy, cystic hygroma, hemangioma, rhabdomyosarcoma, teratoma, lymphoma, papilloma
Endocrine	Thyroid goiter
Traumatic	Laryngeal injury or fracture, thermal injury, upper airway hemorrhage, caustic ingestion
Allergic	Angioedema, anaphylaxis, exercised induced anaphylaxis
Neurologic	Brainstem stem compression, upper motor neuron injury, lower motor neuron injury, tic disorders, multiple sclerosis, postpolio syndrome, multiple system atrophy, myasthenia gravis, Parkinson disease, respiratory spasmodic dysphonia, traction on the recurrent laryngeal nerve, adductor laryngeal breathing dystonia
Pulmonary	Asthma, exercise induced bronchoconstriction, chronic obstructive pulmonary disease, foreign body aspiration, hyperventilation syndrome, pulmonary embolus
Congenital	Laryngomalacia, laryngeal cleft, intrathoracic vascular ring, subglottic stenosis, laryngeal web
Occupational	Inhalation injury

## Disease mechanism and triggers

Vocal cord dysfunction is due to transient obstruction of the upper airway associated with paradoxical adduction (closure) of the vocal folds (cords) and can occur during one or both stages of the respiratory cycle [1].

The larynx functions to provide protection of the lower airway, respiration, and phonation, all of which are regulated partially by involuntary brainstem reflexes. The protective function of the larynx is strictly reflexive, whereas the other two functions can be initiated voluntarily [19]. Pulmonary protection is mediated by the glottic closure and cough reflexes to protect the lower airway from noxious inhaled stimuli and aspiration of foreign material during respiration [16, 19]. The cough reflex is usually initiated by an adverse stimulus triggering one of the many sensory receptors of the larynx [16].

Normally, the vocal cords abduct (open) widely during inhalation, just before the onset of inspiratory flow, reaching a maximum width at mid-inspiration. During exhalation, vocal cord movement varies significantly between individuals but generally adducts between 10 and 40 % of the aperture from end inspiration until approximately two third of vital capacity is expelled [20].

Vocal cord dysfunction is most likely due to laryngeal hyperresponsiveness, with increased sensitivity of the laryngeal sensory receptors and heightened response of the glottic closure and cough reflexes to a number of triggers, which are discussed later [1]. Maschka and colleagues describe a number of organic causes of abnormal vocal fold movement, including primary neurologic disorders, such as brainstem compression, upper motor neuron injury, lower motor neuron injury and movement disorders [21]. Vocal fold paralysis caused by head and neck malignancy, recurrent laryngeal nerve damage during surgery, vocal fold paresis from prolonged intubation, and even idiopathic vocal fold paralysis can all cause abnormal vocal fold movement and symptoms similar to those seen in VCD [18]. However, these organic causes are distinguished by the fact that they do not generally create intermittent paroxysms of vocal cord adduction but rather varying degrees of fairly consistent abnormal movement of the vocal folds.

VCD episodes frequently begin and end abruptly, so specific triggers are not always identified [1, 22, 23]. Self-reported triggers include upper respiratory infections, occupational exposures, talking, laughing, singing, acid reflux, cough, foods, physical exertion, exercise, post nasal drip, weather changes, emotional stressor, odors, strong scents and other airborne irritants [12, 24]. Some patients even report a priming effect where they initially have a single trigger but eventually develop multiple triggers that were previously benign [25, 26]. Christopher and Morris classify triggers for vocal cord dysfunction into exertional, psychological and irritant categories [18].

### Exercise as a trigger

Exertional VCD can be caused by maximal exercise or athletic competitions but can also be seen during routine exercise [18]. Exercise-induced VCD can be seen in many patients who are highly competitive, in elite athletes, and in active duty military personnel who are required to exercise regularly. Exercise was initially recognized as a cause of VCD in 1984 in a 33-year-old female competitive runner who developed wheezing during exercise. She was treated for 10 years for exercise-induced asthma, but upon further evaluation her methacholine challenge testing was negative and her post exercise flow-volume loops showed characteristic flattening of the inspiratory limb [27]. In 1996, McFadden described seven elite athletes who reported a “choking” sensation during exercise but had normal baseline pulmonary function testing (PFTs) and negative bronchoprovocation testing. On spirometry they had the characteristic flattening of the post exercise flow-volume loop [28]. A study of active duty military patients with exertional dyspnea found that 12 % of the patients had VCD triggered by exercise [29].

### Psychological triggers

As evidenced by the initial terminology used to describe VCD, including “hysteric croup”, “Munchausen’s stridor” and “emotional laryngeal wheezing”, initial reports of VCD emphasized the dominant underlying psychological disorders in these patients. It is still thought that psychological stimuli can trigger VCD, including anxiety disorder, stress, depression, somatoform disorder, conversion disorder, psychiatric illness, history of sexual abuse, and mass psychogenic illness [18]. In a case series published in 1983 of five patients with “uncontrolled” asthma and dramatic wheezing all found to have VCD, a psychiatrist performed personality testing prior to any physiologic or laryngoscopic studies and established a psychiatric diagnosis in four of the five patients [8]. In a review by Lacy and McManis in the late 1990s, 45 of 48 patients had a psychiatric condition, including conversion disorder (52 %), major depression (13 %), factitious disorder (10 %), obsessive-compulsive disorder (4 %) or adjustment disorder (4 %) [30]. While some studies have suggested that VCD may be the result of conversion disorder, not all patients with VCD have an underlying psychiatric illness [31–33]. A recent prospective study evaluating psychological disorders in 45 patients with VCD demonstrated a classic conversion profile on Minnesota Multiphasic Personality Inventory-2 testing in 40 % of patients, but 25 % of patients had no evidence of psychopathology [34]. Some investigators actually suggest that depression and anxiety are often seen in these patients as a result of their chronic respiratory illness rather than as the cause of their condition [20, 31, 33].

### Irritant triggers

Irritant triggers can be intrinsic, such as gastroesophageal reflux disease or rhinitis, or extrinsic, including chemical irritants and olfactory and even visual stimuli [18]. One theory of vocal cord dysfunction involves laryngeal hyperresponsiveness and accentuation of the glottic closure reflex caused by these intrinsic or extrinsic triggers [35]. The sensory receptors that mediate the cough and glottic closure reflexes in the larynx, trachea and larger airways can be stimulated directly or indirectly via olfactory nerve stimulation or direct stimulation of sensory nerve endings. This stimulation leads to closure of the vocal folds, and this reflex may be accentuated in patients with VCD [15, 16]. Diseases such as postnasal drip, gastroesophageal reflux disease, pharyngitis, laryngitis and sinusitis can lead to laryngeal inflammation and hyperresponsiveness [36–39].

## Diagnosis and testing

The first step in diagnosis of vocal cord dysfunction involves a careful history and physical exam looking for characteristic features of vocal cord dysfunction. Diagnosis of vocal cord dysfunction can be identified with the use of laryngoscopy, ideally performed after a bronchoprovocation challenge. It can also be suggested by the appearance of the flow-volume loop obtained through spirometry or pulmonary function testing as well as through impulse oscillometry, although the latter is not as readily available [40].

### Assessment of symptoms

Characteristic features of vocal cord dysfunction are described in the clinical presentation section. Table 2 includes a list of relevant questions to discuss with patients to guide diagnosis and differentiate vocal cord dysfunction from other etiologies. Fowler and colleagues recently proposed a 12-item questionnaire, called the VCDQ, as a valid tool for symptom monitoring in patients with VCD. This questionnaire, which incorporates features of many questions listed in Table 2, showed improvement in scores after speech therapy. While this scoring system is new and has not yet been studied in large populations, it may serve as a novel way to assess severity of disease and monitor response to therapy in such patients [41].

**Table 2:** Pertinent questions for evaluation of VCD [41]

1. Do you feel like your symptoms are confined to your throat or upper chest?
2. Do you feel like there is a restriction in your throat or upper chest preventing you from getting air past a certain point?
3. Do you have shortness of breath when breathing in?
4. Do you have a sudden onset of your attacks?
5. Do you a sensation of something in your throat you are unable to clear?
6. Does your voice change when you have an attack?
7. Do you feel your breathing is loud or noisy during attacks?
8. Do specific triggers cause your attacks?
9. Do you feel your symptoms have not been understood correctly?
10. Do you have difficulty with light pressure, such as tight clothes or bending your neck?
11. Are your attacks impacting your social life?
12. Do asthma medications help?
13. Do use of your asthma inhalers sometimes make symptoms worse?
14. Do you ever feel lightheaded or dizzy during attacks?
15. Do you have numbness or tingling in your hands, feet or lips with attacks?
16. When your symptoms start, do you generally cough?

Traister and colleagues developed a useful scoring system to help distinguish VCD from asthma called the Pittsburgh VCD index. This simple, valid and easy to use clinical tool assigns patients a weighted score based on symptoms of throat tightness (score of 4) and dysphonia (score of 2), the absence of wheezing (score of 2) and the presence of odors as a trigger for symptoms (score of 3). A cutoff of ≥ 4 yielded an 83 % sensitivity and 95 % specificity for the diagnosis of VCD. Upon application to a population with known VCD, this scoring system correctly diagnosed VCD in 77.8 % of patients. Of course, since many patients have coexistent VCD and asthma, further diagnostic tests should be performed if a strong suspicion for asthma exists [42].

### Flexible laryngoscopy

**Fig. 1:**
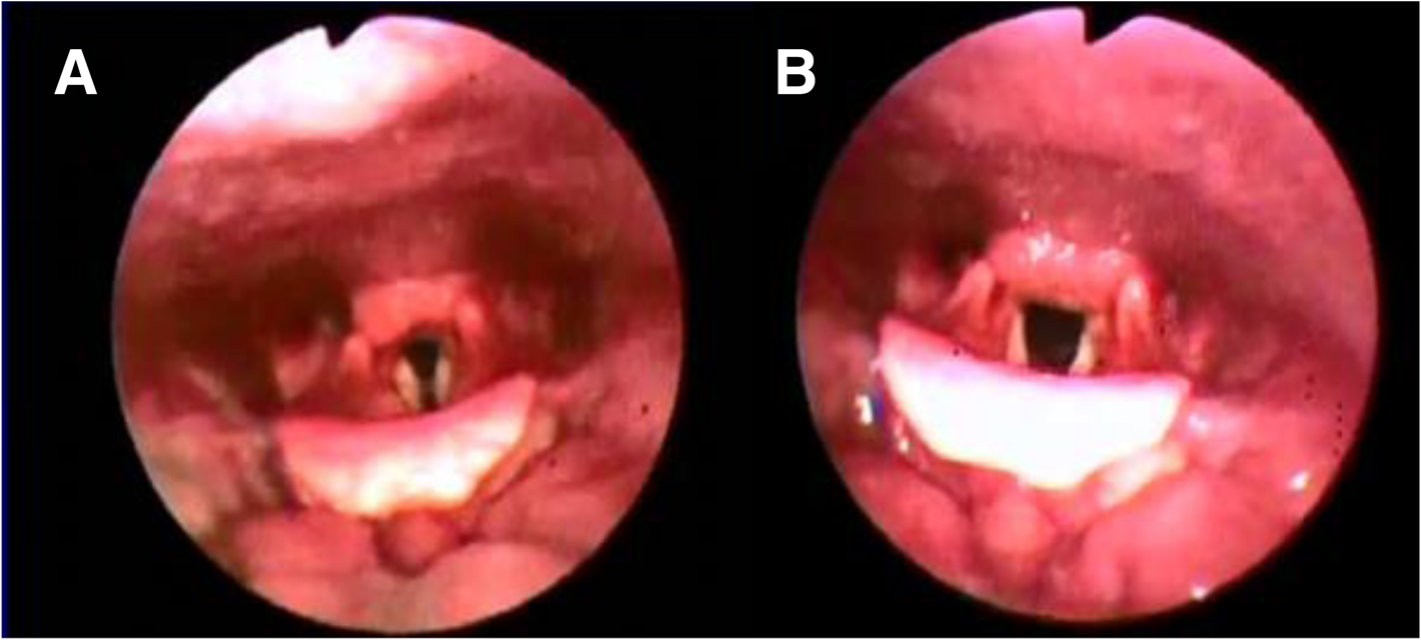
**a** Images taken during laryngoscopy showing paradoxical adduction detected during mid-inspiration in a patient with vocal cord dysfunction **b** Appropriate movement of the vocal cords during mid-inspiration in the same patient following speech therapy

Endoscopic examination with direct visualization of the vocal folds via flexible, transnasal fiber-optic laryngoscopy during an acute attack is the gold standard for diagnosis of VCD [8, 11, 31]. The presence of inspiratory adduction is key to making the diagnosis [18]. Brugman found complete inspiratory vocal fold adduction at mid-inspiration was the most common laryngoscopy finding in 66 % of adult and pediatric patients with known VCD [7]. Figure 1a shows images from the laryngoscopy of a patient with paradoxical vocal fold adduction during mid-inspiration, whereas Fig. 1b shows laryngoscopy images from the same patient following successful speech therapy.

The endoscopic examination of patients with VCD is frequently normal when patients are symptom-free. Patients should be instructed to perform various maneuvers during laryngoscopy including sniff, sequential phonation, normal breathing, panting and repetitive deep breaths in order to fully evaluate vocal cord movement [8, 18, 20]. A forced expiratory and inspiratory vital capacity maneuver that simulates generation of a flow-volume loop may be helpful in discovering abnormal vocal cord movement [18]. VCD occurring exclusively during expiration is uncommon and should not be confused with glottic narrowing that occurs in asthmatics to allow for intrinsic positive end-expiratory pressure (PEEP) [8].

### Bronchoprovocation challenge

Performing a laryngoscopy immediately after a bronchial challenge can help determine whether a patient has asthma, VCD, or both [1]. Patients with VCD often show inappropriate vocal fold movement during inspiration or expiration when laryngoscopy is performed immediately following a bronchoprovocation challenge with methacholine. However, patients who are asymptomatic may show normal vocal fold movement. Therefore, a negative laryngoscopy in an asymptomatic patient does not rule out VCD [15]. Nonetheless, bronchial provocation with methacholine has a high negative predictive value and can be helpful in ruling out the diagnosis of asthma [43]. In patients with a compelling history who fail to react to methacholine, an irritant challenge under close observation to a known trigger or provocation with exercise may be indicated to elicit symptoms [19, 20, 28, 35].

### Pulmonary function testing

**Fig. 2:**
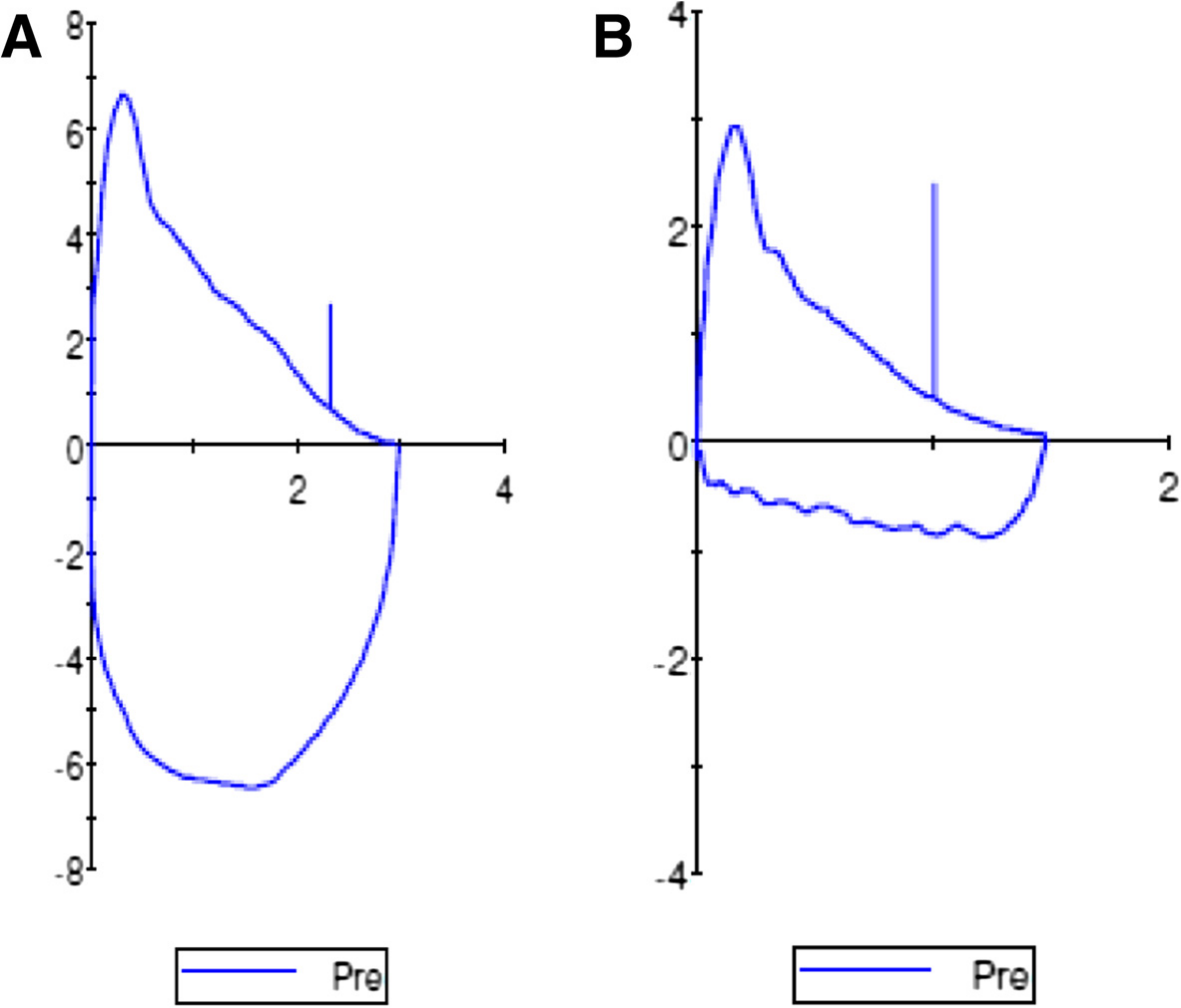
**a** Normal flow volume loop in asymptomatic patient. **b** Example of flattening, early truncation and saw-tooth pattern of inspiratory limb of flow volume loop in a patient with vocal cord dysfunction

A reported characteristic finding in VCD is a highly variable, non-reproducible and abnormally shaped inspiratory loop on spirometry consistent with a variable extrathoracic obstruction, as described by Miller and Hyatt [15, 44]. Morris et al. reviewed 1500 cases of VCD in the published literature and found that 28 % of reported VCD patients had flow-volume loop truncation on spirometry [8]. As seen in Fig. 2, the inspiratory flow loop can show flattening, truncation, and/or saw tooth pattern in patients with VCD, either during an acute VCD attack or even when patients are asymptomatic [15, 16, 20]. Blunting of both inspiratory and expiratory loops, on the other hand, is consistent with a fixed obstruction and should be further evaluated radiographically or endoscopically for a fixed process rather than a functional disorder [20].

Another useful measure in pulmonary function testing is the FEF50/FIF50 ratio, which is usually less than one in normal individuals. In patients with inspiratory VCD, this ratio is usually greater than 1 because truncation of the inspiratory loop reduces the FIF50. For patients with VCD and concomitant expiratory obstruction or comorbid asthma, however, this ratio may be difficult to interpret [8, 16, 45].

### Impulse oscillometry

Komarow and colleagues found that impulse oscillometry (IOS) exhibits a characteristic impedance pattern in patients with VCD, verified by laryngoscopy. This may offer a rapid, noninvasive adjunct to diagnose patients with VCD [40] The main drawback to this type of testing, however, is the impulse oscillometers are not readily available.

## Treatment and management

Despite the typically benign and self-limited episodes, VCD can lead to severe symptoms, the impression of impending respiratory failure, and even emergent intubation or tracheostomy. Management of VCD often requires a multidisciplinary approach involving the primary care physician, pulmonologist, allergist, otolaryngologist, gastroenterologist, neurologist, psychiatrist or psychologist, speech pathologist, and athletic trainer [16, 46].

The management of VCD, especially in the acute setting, requires establishing the correct diagnosis. In the absence of impending respiratory failure, performing a laryngoscopy while symptomatic is rapid, safe and informative in most patients. Once the diagnosis is established, treatment should be aimed at acutely relieving airway obstruction [18]. Asthma medications, including inhaled bronchodilators and corticosteroids, should be used only if the diagnosis is unclear, but patients may have minimal response to them [18]. Once the diagnosis of VCD is confirmed, the first step is to reassure patients that the condition is benign and self-limited. While the use of medications can be attempted, effective long-term therapy requires psychosocial support, speech therapy and even biofeedback.

### Patient education

Patient education is a crucial component of treatment. Knowledge of normal physiology and functional abnormalities causing symptoms can help patients accept their diagnosis and gain control over this disorder. Allowing patients to view their laryngoscopy findings often enhances understanding and acceptance. Patients previously misdiagnosed with asthma instead of VCD should have unnecessary medications discontinued gradually and under the care of their physician [8, 10, 33].

### Medications

Sedation with benzodiazepines has proven successful in some patients, especially when underlying anxiety is a contributing factor [8, 12]. Heliox, a helium/oxygen mixture that leads to a reduction of air density, decreases turbulent flow, and reduces work of breathing, has shown favorable responses in acute VCD episodes and has even demonstrated a sustained response after discontinuation in many cases [10]. An invasive and rarely used treatment modality is laryngeal injection of botulinum toxin type A, which prevents acetylcholine release at nerve endings, leading to chemical denervation and paralysis of the vocal fold in the open position [8, 9]. While this has been successfully used to treat spasmodic dystonia [33], a review by Morris and colleagues in 2006 found only 9 reported cases of botulinum toxin used to treat VCD [8]. Given the risk associated with paralyzing vocal cords in an open position, it should be reserved for patients refractory to all other therapies or those considering tracheostomy [8, 9].

### Speech therapy and psychotherapy

The most common long-term treatment is speech therapy and psychotherapy. Speech therapy consists of a detailed assessment of patient’s symptoms and triggers, followed by a comprehensive treatment that is tailored to the individual patient [17]. Patients are educated about the pathophysiology of VCD, are provided supportive counseling, and are educated about suppression of laryngeal abusive behaviors (i.e., cough and throat clearing), voice therapy, respiratory retraining, and desensitization to specific irritants. They are taught various breathing techniques, known as quick-release techniques, which act to rapidly release the vocal folds from the paradoxical movement responsible for symptoms of VCD. These exercises focus on pursed-lip breathing using abdominal support, with a focus on relaxation. Patients are encouraged to practice this technique with 5 repetitions 20 times per day to assist with laryngeal relaxation and retraining and to ensure that patients can respond automatically when acutely symptomatic [17]. Patient progress can be followed clinically or with more objective approaches, such as the VCDQ described above, which has shown improvement in scores after speech therapy [41]. In addition to speech therapy, patients may benefit from psychological counseling, which lacks a systematic study but may be warranted in those whose VCD is related to an underlying psychiatric condition [33, 47].

### Biofeedback

Biofeedback and hypnosis have also shown some benefit in patients with VCD [47–50]. McFadden and Zawakski found that four of nine patients with exercise induced VCD had rapid resolution of symptoms after basic biofeedback [28]. In another study, the use of hypnosis in 29 VCD patients showed improvement in 31 % and resolution in another 38 % [51].

## Conclusions

Vocal cord dysfunction can be difficult to treat as the condition is often underappreciated and misdiagnosed in clinical practice. Although symptoms often mimic asthma, VCD and asthma have certain distinct clinical features and specific findings on diagnostic studies, which can serve to differentiate the two conditions. Early recognition and accurate diagnosis of vocal cord dysfunction can prevent improper treatment and, therefore, minimize escalated health care costs. The Pittsburgh VCD index and the VCDQ may serve as useful tools to differentiate asthma and VCD and to measure symptom improvement after treatment. While speech therapy is currently the mainstay of treatment, biofeedback and pharmacotherapy have been successful in select patients. Since intrinsic and extrinsic triggers can exacerbate laryngeal hyper-responsiveness, a focus on minimizing such triggers can also serve to improve symptoms. Further studies are needed to investigate novel therapies for refractory patients.

## Abbreviations

VCD: Vocal cord dysfunction
PFT: Pulmonary function testing
FVL: Flow volume loops
VCDQ: Vocal cord dysfunction questionnaire
PEEP: Positive end expiratory pressure
IOS: Impulse oscillometry
